# Genomic diversity contributes to the neuroinvasiveness of the Yellow fever French neurotropic vaccine

**DOI:** 10.1038/s41541-021-00318-3

**Published:** 2021-04-26

**Authors:** Florian Bakoa, Christophe Préhaud, Guillaume Beauclair, Maxime Chazal, Nathalie Mantel, Monique Lafon, Nolwenn Jouvenet

**Affiliations:** 1 Unité de Neuroimmunologie Virale, Institut Pasteur, Paris, France; 2 Research and External Innovation Department, Sanofi Pasteur, Marcy L’Etoile, France; 3 Sorbonne Université, Collège doctoral, Paris, France; 4 Unité de Signalisation Antivirale, CNRS UMR 3569, Institut Pasteur, Paris, France; 5 Institut de Biologie Intégrative de la Cellule, UMR9198, Équipe Autophagie et Immunité Antivirale, Faculté de Pharmacie, Châtenay-Malabry, France

**Keywords:** Virology, Vaccines, Live attenuated vaccines

## Abstract

Mass vaccination with the live attenuated vaccine YF-17D is the current way to prevent infection with Yellow fever virus (YFV). However, 0.000012–0.00002% of vaccinated patients develop post-vaccination neurological syndrome (YEL-AND). Understanding the factors responsible for neuroinvasion, neurotropism, and neurovirulence of the vaccine is critical for improving its biosafety. The YF-FNV vaccine strain, known to be associated with a higher frequency of YEL-AND (0.3–0.4%) than YF-17D, is an excellent model to study vaccine neuroinvasiveness. We determined that neuroinvasiveness of YF-FNV occured both via infection and passage through human brain endothelial cells. Plaque purification and next generation sequencing (NGS) identified several neuroinvasive variants. Their neuroinvasiveness was not higher than that of YF-FNV. However, rebuilding the YF-FNV population diversity from a set of isolated YF-FNV-N variants restored the original neuroinvasive phenotype of YF-FNV. Therefore, we conclude that viral population diversity is a critical factor for YFV vaccine neuroinvasiveness.

## Introduction

The Yellow fever virus (YFV) belongs to genus *Flavivirus*, which gathers ~70 viruses, including the pathogenic dengue virus (DENV) or Zika virus (ZIKV). Flaviviruses are small enveloped viruses carrying a single-stranded, positive-sense RNA genome that codes for three structural proteins, core (C), membrane (prM/M), and envelope (E), and seven nonstructural (NS) proteins^1^. YFV is endemic in tropical areas of Africa, Central, and South Americas^2^. It is transmitted mainly to nonhuman primates and humans by mosquitoes of the *Haemogogus*, *Sabethes*, and *Aedes* species. Due to poor vaccine coverage and vaccine shortage, YFV regularly resurges in the African and South American populations, as illustrated by recent outbreaks in Brazil and equatorial Africa^3–5^. YFV triggers a spectrum of clinical symptoms including minor symptoms up to acute febrile viscerotropic illness that may cause hepatitis (jaundice), renal failure, hemorrhagic fever, and death. Around 130,000 severe cases occur each year resulting in up to 70,000 deaths^2^.

Yellow fever is controlled by the use of live-attenuated vaccines that have been developed in the 1930s^6,7^. Among these, the French neurotropic vaccine (FNV) was generated by 260 passages of the French viscerotropic virus (FVV) through serial mouse brain passages^7–10^. It was widely administered in French-speaking Africa during 40 years. However, its use was discontinued in 1982 due to a high level of post-vaccinal complications, including encephalitis in children, and evidence of reversion to a virulent phenotype. Today, the YF-17D vaccine developed by passaging the blood of a human patient in rhesus macaques and, later in mouse and chicken embryo tissues, is the only one in use^8^. More than 587 million doses have been administered from 1939 to 2016^11^. A single dose confers protective long-term immunity^12,13^. During the attenuation process, the virus has lost its viscerotropic properties, which account for the major disease manifestations of YF in primates^8,14^. The vaccine is well tolerated and post-vaccination complications are so infrequent that the vaccine can safely be given to 9-month-old children. Nevertheless, severe adverse events can occur after YF vaccination. These are anaphylactic reactions, vaccine associated viscerotropic disease (YEL-AVD), or vaccine associated neurotropic diseases (YEL-AND). YEL-AND is characterized by encephalomyelitis or meningoencephalitis. Their frequency is higher than those of YEL-AVD (0.8 and 0.3 per 100,000 doses distributed for YEL-AND and YEL-AVD, respectively^15^, as calculated by Lindsey et al.^16^, by consulting the US Vaccine Adverse Event Reporting System on the 2007 to 2013 period). Their rate increases with age (2.2 per 100,000 vaccines for patient over 60 years old, compared to 0.8 per 100,000 vaccines younger than 60)^17^. Origin of YEL-AND is not clear^14^. One hypothesis is that they could result from the appearance of variants during the preparation of the vaccine seed lots. Such variants would have acquired the possibility of entering the brain (neuroinvasiveness), propagating into the nervous tissues (neurotropism), and causing neurotoxic reactions (neurovirulence). Such a possibility is supported by the isolation from a fatal case of vaccine associated viral encephalitis of a neuroinvasive/neurotropic and neurovirulent variant which sequence was distinct from those of the vaccine strain (YF-17D-204)^18^. Understanding the factors responsible for neuroinvasion, neurotropism, and neurovirulence of the YF vaccine is critical for comprehensive monitoring of its safety. In particular this information may improve quality control assays performed in nonhuman primates to check for the absence of virulence of YF vaccines seed lots^19,20^. In addition, a better knowledge of the YEL-AND origin might also identify new means to perform quality controls of YF vaccines.

YF variants could gain access to the central nervous system (CNS) through the bloodstream. Early study^21^ and more recent^22^ work support the idea that YF-17D disseminates mainly through the blood to infect the CNS. By using a 3D-human brain–blood interface model, the BBB-Minibrain, that assembles endothelial cells monolayer forming a BBB with a tri-culture of differentiated neurons, astrocytes, and microglia, our group demonstrated that YF-FNV vaccine contains few viral particles which are able to cross the BBB of the device, and to replicate in the Minibrain^23^. Compared to YF-17D, the amount of neuroinvasive virions crossing the BBB was much higher in YF-FNV samples. Thus, studying the passage of the YF-FNV vaccine strain through an in vitro model of human BBB (mBBB) consisting of human endothelial cells seems relevant to understand better the molecular and genetic determinants of YEL-AND. Our goal here was to isolate neuroinvasive virions obtained by in vitro passage of the mBBB, to characterize their genome by next generation sequencing (NGS) technique and to study the mechanisms by which they cross the mBBB.

## Results

### YF-FNV, but not YF-17D, crosses efficiently the mBBB and infects brain endothelial cells

**Fig. 1:**
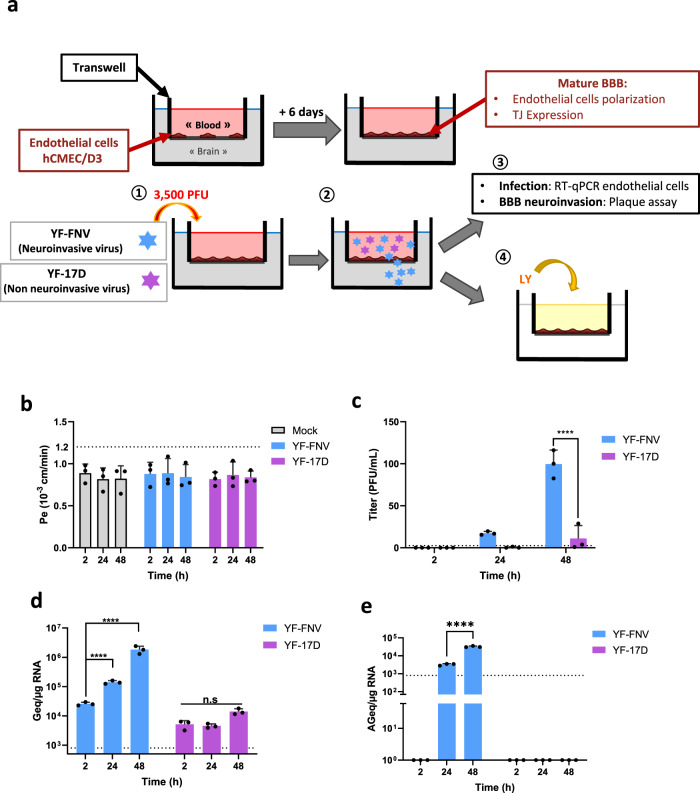
YF-FNV, but not YF-17D, crosses and infects the brain endothelial cells of the BBB. **a** Schematic diagram of the mBBB and description of the experiments. hCMEC/D3 cells were grown for 6 days on the porous filter of a Transwell™ insert to mimic a mature BBB characterized by endothelial cells polarization and formation of thight junctions (TJ). The transwell is fitted into one well of a 24 wells cell culture plate. The endothelial cell layer separates two compartments: the (upper compartment mimicking the “blood” side of the mBBB) and (lower compartment mimicking the “brain” side). (1) The upper compartment is inoculated with 3500 PFU (MOI 0.007) of the viruses (YF-FNV or YF-17D). (2) At different times postinoculation, the neuroinvasive particles (blue stars) cross the BBB unlike the non-neuroinvasive (purple stars). (3) The passage through the BBB of neuroinvasive virions is measured by viral titration of the lower compartment medium. Neuroinvasive virions can be plaque-purified by plaque assay for further characterization, while virus replication in endothelial cells is measured by RT-qPCR. (4) Functional integrity of the BBB is controlled by quantifying the transport of the small fluorescent molecule Lucifer Yellow (LY). (**a**–**e**) Comparison of neuroinvasiveness of the two vaccine strain through the mBBB and of endothelial cell infection at different times postinoculation of the upper compartement. **b** The physiological status of the BBB was tested by quantifying LY transport at 2, 24, and 48 h post virus inoculation (hpi) or mock-inoculation (NI). The threshold limit for a mature BBB with a proper physiological status was set at a permeability of 1.2 × 10^−3^ cm/min (dotted line). **c** The amounts of infectious particles released in the lower chamber at 2, 24, and 48 hpi were quantified by viral titration and expressed as plaque forming unit (PFU/mL). **d**, **e** The relative amounts of genomic (**d**) and antigenomic RNA (**e**) of YF-FNV and YF-17D present in endothelial cells were determined by RT-qPCR analysis. Amounts of viral RNA were expressed as genome equivalents (Geq) or antigenome equivalents (AGeq) per microgram of total cellular RNA. Statistical test: Two-way ANOVA with post hoc Sidak’s test (n.s. not significant; *p < 0.05; ****p < 0.0001). Data in all bar graphs were means ± SD of three independent experiments (*N* = 3) performed in triplicate (*n* = 3). Dashed lines indicate the limit of detection.

The in vitro mBBB we used here is composed of human brain microvascular endothelial cells (hCMEC/D3), a widely-used model for in vitro studies of the BBB, as they retain important BBB characteristics, like expression of efflux transporters^24^ and tight junction proteins (Supplementary Fig. 1a), and as they form a steady polarized barrier when cultured on a permeable membrane (Supplementary Fig. 1b, c). The upper compartment (luminal) mimics the blood vessel, whereas the lower compartment (abluminal) corresponds to the brain side (Fig. 1a). After inoculation of the upper compartment with a viral stock containing both neuroinvasive and non-neuroinvasive virions, only the neuroinvasive subpopulation (blue stars) will cross the mBBB, and reach the lower compartment, while the non-neuroinvasive subpopulation (purple stars) of the viral stock will remain in the upper compartment of the device. The integrity of the mBBB physiological status is determined by measuring the permeability of each device by checking for the passage of the fluorescent molecule LY. The stability of the mBBB integrity was evaluated using P_e_ measurements at day 5, 6, and 7 post-seeding (Supplementary Fig. 1b). Assessement of TEER was used to validate further the mBBB tightness (Supplementary Fig. 1c). TEER values were around 38 and 53 Ω/cm^2^ (Supplementary Fig. 1c), which is in agreement with previously reported TEER values of hCMEC/D3 cells^25^.

The capacity of YF-17D and YF-FNV to cross the mBBB were compared at three time points postinoculation (2, 24, and 48 hpi). Upper compartment of mBBB devices were inoculated with 3500 PFU of each vaccine strains (i.e., which represents a MOI of 0.007). Such a low MOI was chosen in order to test the mBBB crossing of the viruses without disturbing its homeostasis. Indeed, endothelial cell barriers inoculated with YF-FNV or with YF-17D exhibited no change in paracellular permeability at these three time points, as measured by LY paracellular transport (Fig. 1b). Release of infectious viral particles in the lower compartment was titered by plaque assays on Vero cells (Fig. 1c). At 24 hpi, one or zero infectious YF-17D PFU/mL was detected into the lower compartment, whereas an average of 13 infectious YF-FNV PFU/mL were detected. At 48 hpi, around 90 infectious YF-FNV PFU/mL were released, 10-times more than the number of YF-17D infectious particles crossing the mBBB at the same time (Fig. 1c). These data confirm our previous observation using the BBB-Minibrain^26^ and showing that the size of the subpopulation able to cross the mBBB in the YF-FNV viral stock (this population will be referred as YF-FNV neurotropic (YF-FNVn)) is much larger than those present in the YF-17D stock.

To investigate the ability of viruses to cross a disrupted mBBB, scratch-wound assays or mannitol treatment were used. Mannitol is known to transiently perturbate cellular junctions^27,28^. In all, 3500 PFU of YF-17D were deposited onto the mBBB, disrupted or not. As expected^27,28^, both the mannitol treatment and the cell wounding induced a significant increase in LY permeability (Supplementary Fig. 2a). Release of infectious YF-17D particles in the lower compartment 24 h post-viral inoculation was quantified by plaque assays on Vero cells. Around 2.5 PFU/mL were released in the basal medium of the control mBBB, whereas on average 10 and 36 PFU/mL were collected in the basal medium of the mannitol-treated mBBB and wounded samples, respectively (Supplementary Fig. 2b). Therefore, alterations of the mBBB enhanced crossing of viruses through the hCMEC/D3 cell monolayers. These experiments also revealed that the amount of virions crossing the mannitol-treated and wounded mBBB were only around 0.42 and 1.5%, respectively, which suggests that passage of viruses through the mBBB is an active and selective process.

Viral replication in mBBB cells was assessed by measuring the quantity of intracellular positive- (G: genome) and negative-strand (AG: anti-genome) viral RNA over-time by RT-qPCR. To validate the methodology, we performed experiments in hCMEC/D3 cells infected with UV-inactivated viruses. Cells treated with lycorine, an inhibitor of West Nile virus and YFV replication^29^, were included in the analysis. Viral RNA copy number detected at 2 hpi represented the input of viral material (Supplementary Fig. 3). YF-FNV input contained more positive viral RNAs than YF-17D inoculum (Supplementary Fig. 3a). A genuine above-background negative-strand RNA signal was detected at 2 hpi in one YF-17D sample and in three YF-FNV samples (Supplementary Fig. 3b). Since the same MOI was used for the two viruses, detection of positive- and negative-strand viral RNA at 2 hpi suggests that a greater quantity of noninfectious material is present in YF-FNV inoculum than in YF-17D’s. Negative strand RNAs may be packaged into virions as double-stranded RNA molecules^30^. During flavivirus replication, multiple growing nascent positive strand RNAs are synthesized on one negative-strand template at a time^31^. It was thus expected to detect more copies of positive strand RNAs than negative ones. As expected, UV-treated viruses were unable to replicate (Supplementary Fig. 3). All UV-treated YF-17D RNAs were degraded at 48 hpi while some positive-strand YF-FNV RNA resisted degradation (Supplementary Fig. 3). Only one UV-treated YF-FNV sample contained negative strand RNAs (Supplementary Fig. 3b). At 48 hpi, cells infected with both viruses and treated with lycorine produced around 1- to 2-log less positive-strand viral RNAs than mock-treated samples (Supplementary Fig. 3a). Negative-strand viral RNA was undetectable in lycorine-treated YF-17D infected cells (Supplementary Fig. 3b). In YF-FNV-infected cells treated with the inhibitor, the abundance of negative strand viral RNA was similar at 48 hpi than at 2 hpi (Supplementary Fig. 3b). These data validate the selectivity and strand-specificity of either RT-PCR setup.

Infection of mBBB with YF-FNV resulted in a significant increase of viral genome copy numbers from 2 to 48 h (Fig. 1d). By contrast, the amount of intracellular positive-strand RNA of YF-17D did not significantly increase overtime (Fig. 1d). Negative strand RNAs of YF-FNV accumulated in infected cells, confirming the ability of this viral strain to replicate in hCMEC/D3 cells while YF-17D negative strand RNAs was not detected in mBBB (Fig. 1e).

Collectively, these data showed that, in contrast to YF-17D, YF-FNV replicates efficiently in hCMEC/D3 cells without increasing the permeability of the cellular monolayer over the course of infection. This indicates that intrinsically YF-FNV, but not YF-17D, may possess the ability to cross the mBBB and to replicate in endothelial cells.

### YF-FNV crosses the endothelial barriers prior to the onset of viral replication

**Fig. 2:**
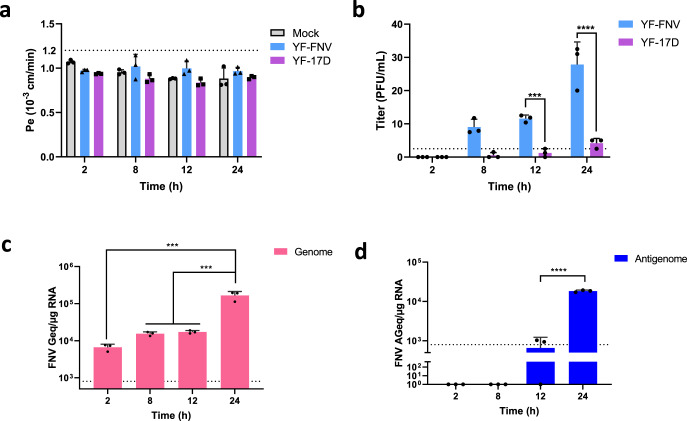
YF-FNV crosses the endothelial barrier prior to the onset (24 hpi) of viral replication in the endothelial cells. The upper compartment of mBBB were inoculated with YF-FNV or YF-17D (3500 PFU). **a** Quatity control of BBB permeability. **b** Transport of infectious particles through the BBB was measured at earlier times (8 and 12 hpi) than the 24 hpi as previously assayed in Fig. 1, to determine whether endothelial cells infection by YF-FNV is a prerequisite for BBB passage. The amounts of infectious particles (PFU/mL) released in the lower chamber were assessed at 2, 8, 12, and 24 hpi by plaque assay. **c**, **d** FNV multiplication in endothelial cannot be detected before 12 h after virus inoculation of the upper compartment of the mBBB. The relative amounts of cell-associated genomic and antigenomic RNA were determined by RT-qPCR analysis. Amounts of viral RNA were expressed as genome equivalents (Geq, **c**) or antigenome equivalents (AGeq, **d**) per µg of total cellular RNA. Statistical analysis: Two-way ANOVA with post hoc Sidak’s test (**p < 0.01; ***p < 0.001 ****p < 0.0001). Data in all bar graphs were means ± SD of three independent experiments (*N* = 3) performed in triplicate (*n* = 3). Dashed lines indicate the limit of detection.

We investigated whether the release of infectious YF-FNV virions from the basolateral membrane of the endothelial barrier was exclusively dependent on the ability of the virus to replicate in hCMEC/D3 cells. We assessed the ability of the two vaccine strains to cross the cell monolayer overtime. Similar protocol and quality control of mBBB than those described in Fig. 1 were followed, but analysis were performed at earlier times (2, 8, and 12 hpi) to assess what happens before the end of one viral cycle of infection (24 hpi). Endothelial barriers inoculated with YF-FNV or with YF-17D exhibited no change in paracellular permeability as measured by LY paracellular transport (Fig. 2a). Consistent with our previous results (Fig. 1), very little or no infectious YF-17D particles were detected into the lower chamber at these four time points, reaching a maximum of 5 PFU/mL at 24 hpi (Fig. 2b). By contrast, infectious YF-FNV particles were released in the basal compartment of the culture starting as early as 8 hpi and increased overtime to reach an average of 28 PFU/mL at 24 hpi (Fig. 2b). To determine whether YF-FNV was replicating in hCMEC/D3 cells at 8 and 12 hpi, production of negative- and positive-strand intracellular viral RNA was analyzed by RT-qPCR (Fig. 2c–d). The analysis showed that the amount of plus-strand viral RNA did not vary significantly during the first 12 h of infection (Fig. 2c). At 24 hpi, this quantity reached around 2 × 10^5^ copies of genomic RNA, which is ten times more than at 12 hpi (Fig. 2c). At 24 hpi, an average of 1.8 × 10^4^ copies of minus-strand viral RNA were detected, which is 20 times more than at 12 hpi (Fig. 2d). These observations indicate that production of YF-FNV RNAs occurs between 12 and 24 h in hCMEC/D3 cells.

Together, these data suggest that YF-FNV could cross the endothelial barriers prior to the onset of viral replication.

### Characterization of the YF-FNVn population sequence

**Fig. 3:**
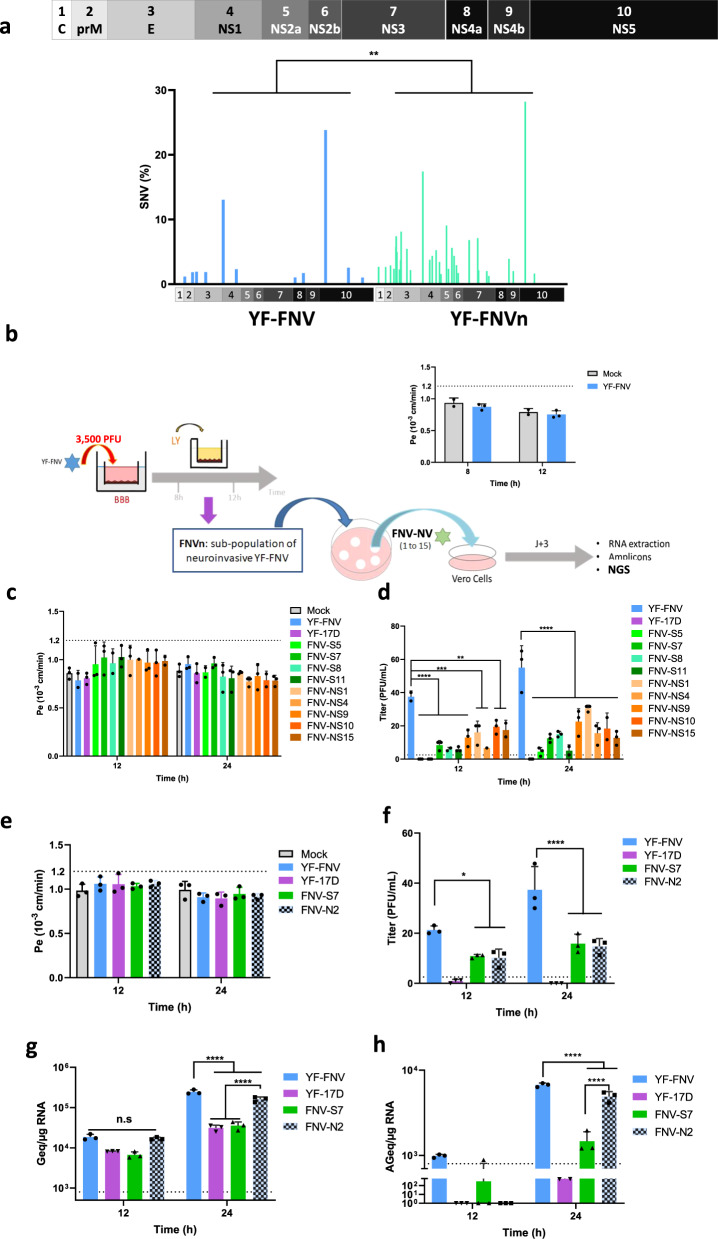
Neuroinvasive variants exhibit a reduced ability to cross the endothelial barrier. **a** A schematic representation of YFV genome was shown where YF genes are numbered (from 1–10). The YF-FNVn population corresponds to the fraction of YF-FNV virions which crossed the BBB and which were collected at 8 or at 12 hpi before being amplified by one passage on Vero cells for 4 days. The cell-associated viral RNA was isolated and processed for NGS analysis to determine the frequencies of single nucleotide polymorphism (SNV) of YF-FNV (blue) and YF-FNVn (green). Only SNV of more than 1% were considered in the analyses of population variability by the Mann–Whitney *U*-test (**p < 0.01). **b** Experimental scheme followed for the isolation by the plaque assay technique of 15 individual viruses from the neuroinvasive subpopulation of YF-FNV (YF-FNVn), their amplification by one passage on Vero cells, and their genomic characterization by NGS. The BBB quality control was tested 8 and 12 hpi. **c**–**d** The neuroinvasiveness of nine variants showing mutations in virus structural proteins (FNV-S5, 7, 8, and 11) or non-structural proteins (FNV-NS1,4, 9,10, and 15) was assayed on BBB which integrity was controlled. **c** The upper compartment was mock inoculated (NI) or inoculated with 2500 PFU of the nine variants, of YF-FNV and of YF-17D (these two viruses were used as positive and negative of controls of neuroinvasiveness). **d** Passage of the BBB was assayed at 12 and 24 hpi by measuring the amounts of infectious particles (titer expressed as PFU/mL) released in the lower compartment. **e**–**f** The impact of viral diversity on FNV neuroinvasiveness was assayed by comparing the efficacy of BBB passage by FNV (high diversity) and two FNV variants, FNV-N2 and FNV-S7 (lower diversity) of distinct genetic diversity. YF-17D was used as a negative control of neuroinvasiveness. The upper compartment of the BBB device was inoculated with 3500 PFU of YF-FNV, YF-17D, FNV-S7, and FNV-N2. **e** Quality control of the BBB permeability. **f** Passage of the BBB assayed at 12 and 24 hpi by measuring the amounts of infectious particles (titer expressed as PFU/mL) released in the lower compartment, indicate that the rate of neuroinvasiveness of FNV-N2, is still lower than those of FNV, suggesting that a polyclonal population of YF-FNV is more neuroinvasive than a clonal population. **g**–**h** The relative amounts of cell-associated genomic and antigenomic viral RNA were determined by RT-qPCR analysis at 12 and 24 hpi. Amounts of viral RNA were expressed as genome equivalents (Geq) or antigenome equivalents (AGeq) per microgram of total cellular RNA. Statistical analysis: Two-way ANOVA with post hoc Sidak’s test (n.s. not significant; *p < 0.05; ****p < 0.0001). Experiments were performed once in triplicate (*N* = 1, *n* = 3) or three times in triplicate (*N* = 3, *n* = 3) and are represented as means ± SD. Dashed lines indicate the limit of detection.

Our goal was to isolate neuroinvasive virions obtained by passage of mBBB and to characterize their genome by NGS. RNA viruses can comprise a cloud of related viruses known as a quasispecies, or viral swarm^32–34^. This phenomenon is a direct result of the low fidelity of the viral RNA-dependent RNA polymerase, which lacks an exonuclease proofreading function resulting in error-prone replication. YF-17D RNA polymerase was shown to exhibit a relatively high fidelity leading to limited evolution in vitro and in vivo^35,36^. NGS analysis of vaccine strains has revealed that YF-FNV is more genetically diverse than YF-17D^37,38^. We tested whether YF-FNV greater diversity could contribute to its enhanced ability to cross the endothelial cell barrier prior to replication. At first, the variability of the vaccine strain before (YF-FNV-P4, a stock of YF-FNV obtained after four passages on Vero cells) and after (YF-FNVn) crossing the endothelial barriers was determined by NGS. As shown in the diagram of Fig. 3a, YF-FNVn exhibited a greater genetic diversity than the initial YF-FNV population.

### Selection of neuroinvasive plaque purified variants

To characterize further the YF-FNVn subpopulations, we isolated neuroinvasive virions and sequenced their genome by NGS. hCMEC/D3 cell barriers were inoculated with 3500 PFU from a stock of YF-FNV-P4. In order to analyze the viral population that crossed the endothelial barrier prior to the onset of replication, the viruses released in the lower compartment were harvested at 8 and 12 hpi (Fig. 3b). Since very little viruses cross the endothelial barrier at this time point (Fig. 2), an amplification step of YF-FNVn was performed on Vero cells. One could worry that this amplification step on Vero cells modify the viral genetic diversity. This is unlikely since no difference in the diversity of the viral populations was observed by NGS analysis when we determined the consensus sequence of the YF-FNV population after three passages in Vero cells (Supplementary Fig. 4 and Supplementary Table 1). Thus, despite the low fidelity of the YF-FNV polymerase^37^, three passages on Vero cells had no or very little impact on the diversity of the viral population. The YF-FNV strain used here, which was generated by passaging an original vaccine vial one time in Vero cells, appeared more stable than a plaque-purified variant isolated from another FNV stock, the FNV-IP^37^. This could be due to the initial heterogeneity of the two YF-FNV populations.

To understand the neuroinvasiveness of YF-FNV, we decided to focus on the mutations in the translated region of the viral genome. The comparison of the sequence of YF-FNV and YF-FNVn identified 106 synonymous and 36 non-synonymous mutations (Fig. 3a and Table 1). These differences were scattered along the genome. Among all the variants, the mutations were mostly localized on the E protein (ten mutations) and in the NS1 protein (seven mutations). All the mutations in the E protein had a frequency below 8.1% while the mutations frequency in the NS1 were between 3.4 to 9%, at the exception of one mutation (17.1%) (Fig. 3a and Table 1). YF-FNV-P4 and YF-FNVn have thus identical consensus sequences. Nevertheless, the comparison of the global genetic diversity between YF-FNV-P4 and YF-FNVn revealed statistically significant changes in non-synonymous single nucleotide variants (SNVs) after passage of the endothelial barrier (Fig. 3a). These data suggest that here the ability of YF-FNV to cross the mBBB is probably due to the combination of several variants in the initial YF-FNV-P4 stock and not to the presence of a unique variant.

**Table 1:** Single nucleotide frequency in the YF-FNVn population.

Protein	nt position	AA position	AA reference	AA mutation	FNVn frequency (%)	YF-FNV P4 frequency (%)
**C**	237	40	P	L	2.7	
**prM**	620	47	Y	H	2.7	
**M**	882	45	T	I	2.9	
**E**	1055	28	D	N	2.4	
	1172	67	H	Y	5.7	
	1184	71	N	D	7.4	1.9
	1221	83	A	V	4.9	
	1351	126	S	R	2.3	
	1419	149	Q	P	3.7	
	1436	155	D	N	2.7	
	1446	158	T	I	8.1	
	1755	261	A	V	5.4	
	1947	325	P	L	2.2	
**NS1**	2607	52	E	G	9.0	
	2610	53	G	E	17.4	13.1
	2975	175	Y	H	3.4	
	2982	177	I	K	3.8	
	3110	220	D	N	4.4	
	3318	289	G	D	5.2	
	3501	350	V	A	3.4	2.3
**NS2a**	3584	26	G	Q	1.5	
	3878	124	K	E	9.1	
	3989	161	A	T	2.4	
	4169	221	F	V	5.6	
**NS2b**	4289	37	I	L	4.4	
	4416	79	S	N	2.9	
	4506	109	I	T	1.7	
**NS3**	5094	175	E	A	6.8	
	5549	327	S	G	7.1	
	5595	342	T	M	2.1	
	6033	488	H	R	2.0	
	6150	527	M	K	1.3	
**NS4b**	7225	113	I	M	3.9	
	7457	191	F	L	2.0	
**NS5**	8100	155	V	A	28.2	23.8
	8588	318	A	T	1.6	

The YF-FNVn population corresponds to the virions of YF-FNV which crossed the BBB at 8 or at 12 hpi before being amplified by one passage on Vero cells for 4 days. The cell-associated viral RNA was isolated and processed for NGS analysis. The frequencies of single nucleotide polymorphism (SNV) of YF-FNVn is indicated as well as the amino acid changes in the coding region. Only SNV of more than 1% were considered. The frequency of SNV obtained in the parental FNV population is indicated in the last column.

To isolate and identify the neuroinvasive variant(s) (NV) that could contribute to the ability of the YF-FNV-P4 population to cross the endothelial cell barrier without replicating, virions released in the lower compartment of the mBBB were harvested at 8 hpi and 12 hpi and were plaque purified on Vero cells. Four days after inoculation, fifteen individual plaque-purified variants, each generated by an individual virus, were picked up and amplified on Vero cells prior to NGS analysis (Fig. 3b). The NGS analysis revealed that each of the ten non-synonymous variants exhibited a homogeneous nucleotide sequence with all identified SNVs representing at least 78% of the nucleotides sequenced in one position (Table 2). These data validate the selection of these variants in the Transwell passage and the plaque purification. The NGS analysis also revealed three categories of clones. The first category comprises variants carrying at least one non-synonymous mutation that translates into an amino acid change in a structural protein, which we termed “structural variants (S)” (FNV-S5, -S7, -S8, and -S11) (Table 2). The second category are the variants carrying mutations that lead to at least one amino acid change in non-structural proteins exclusively (“non-structural variants, NS”) (FNV-NS1, -NS4, -NS9, -NS10, -NS13, and -NS15). Finally,“silent variants” exhibited exclusively silent (neutral, N) mutations that do not impact the viral proteins sequences (FNV-N2, -N3, -N6, -N12, and -N14) (Table 2).

**Table 2:** Characterization of 15 neuroinvasive plaque purified variants from the neuroinvasive YF-FNVn subpopulation.

	Name	Protein	nt position	AA position	AA reference	AA mutation	Frequency (%)	Frequency in YF-FNV (%)
**Structural variants**	FNV-S5	M	789	14	T	N	96.2	<1
		NS1	3377	309	P	S	95.5	<1
	FNV-S7	E	1775	268	T	A	92.3	<1
	FNV-S8	E	1086	38	K	R	99.9	<1
		NS5	8100	155	V	A	99.9	23.8
	FNV-S11	M	765	6	T	M	97.9	<1
**Non Structural variants**	FNV-NS1	NS5	8100	155	V	A	97.6	30.0
	FNV-NS4	NS1	2610	53	G	E	88.4	13.1
	FNV-NS9	NS1	2610	53	G	E	99.5	13.1
	FNV-NS10	NS1	2610	53	G	E	83.8	13.1
		NS5	9324	563	K	R	78.5	2.5
	FNV-NS13	NS1	2610	53	G	E	92.8	13.1
	FNV-NS15	NS1	3318	289	G	D	96.6	2.3
**Silent variants**	FNV-N2	**No mutation**						
	FNV-N3	**No mutation**						
	FNV-N6	**No mutation**						
	FNV-N12	**No mutation**						
	FNV-N14	**No mutation**						

Fifteen of the plaque purified NV which crossed the BBB at 8 h or 12 hpi were analysed by NGS. Three groups of variants were characterized: the first one comprises variants carrying at least one non-synonymous mutation in a structural protein, which we termed “structural variants” (FNV-S5, -S7, -S8, and -S11), the second one are the variants carrying mutations that lead to at least one amino-acid change in non-structural proteins exclusively (non-structural variants) (FNV-NS1, -NS4, -NS9, -NS10, -NS13, and -NS15), finally, “silent variants” (FNV-N2, -N3, -N6, -N12, and -N14) exhibited exclusively silent (neutral) mutations that do not impact the viral proteins sequences.

### Plaque variants exhibit a reduced ability to cross the endothelial barrier

The ability of nine of the structural and non-structural variants to cross the mBBB model was analyzed at 12 and 24 hpi (Fig. 3c, d). Here, hCMEC/D3 cells were inoculated with 2500 PFU of viruses (MOI = 0.005). Quantification of the paracellular transport of LY showed that none of the selected variants altered the endothelial permeability at these two time points (Fig. 3c). Viruses released in the lower chamber were titrated by plaque assays on Vero cells. Surprisingly, all variants crossed the endothelial barrier less efficiently than YF-FNV at these two time points (Fig. 3d). When cells were infected with FNV-S5, which carries one mutation in the viral protein M and one in NS1, no infectious particles were detected in the lower chamber at 12 hpi, whereas all the other variants released less than 20 PFU/mL in the lower chamber (Fig. 3d). Since YF-FNV is more genetically diverse than the isolated variants, one can envisage that viral diversity favors the passage of the endothelial barrier.

To investigate this possibility, we compared the neuroinvasiveness of four viruses with distinct genetically diversities: FNV-N2, FNV-S7, YF-FNV, and YF-17D (Fig. 3e, d). FNV-N2, which possesses only silent mutations and thus has an amino acid sequence identical to the consensus sequence of the parental YF-FNV, exhibits a relatively low genetic diversity (Table 2). FNV-S7 was included in the analysis as a poorly diversified variant that exhibited a single mutation in the structural gene E (Table 2). YF-FNV and YF-17D strains were used as positive and negative controls of neuroinvasiveness in these experiments. mBBBs were inoculated with 3500 PFU of the four viruses. Cells and bottom chamber media were collected at 12 and 24 hpi. None of the viruses disrupted the integrity of the hCMEC/D3 cell monolayers (Fig. 3e). Significantly less infectious particles were recovered from the lower chamber of cells infected with FNV-N2 and FNV-S7, as compared to YF-FNV, both at 12 and 24 hpi (Fig. 3f). These data suggest that for FNV background, genetic diversity promotes neuroinvasiveness.

FNV-N2 replicated in endothelial cells with a similar efficiency than YF-FNV, both producing around 2 × 10^5^ copies of genomic viral RNA and 6 × 10^3^ copies of minus strand RNA, per μg of total RNA at 24 hpi (Fig. 3g, h). These amounts represented significant increase as compared to the viral RNA detected at 12 hpi (Fig. 3g, h). The results indicate that genomic diversity is not a determining factor for multiplication in endothelial cells. By contrast, the amount of plus and minus RNA did not vary significantly between 12 and 24 hpi in cells infected with FNV-S7 (Fig. 3g, h). Thus, the unique mutation present in the E protein (T268A) of FNV-S7 significantly impairs viral multiplication in endothelial cells.

Together, these data support the hypothesis that the genetic diversity of the parental strain YF-FNV—and not the mutation that impact the replication in the endothlelial cells— plays a role in the passage of the endothelial barrier.

### The genetic diversity of YF-FNV contributes to its neuroinvasiveness

**Fig. 4:**
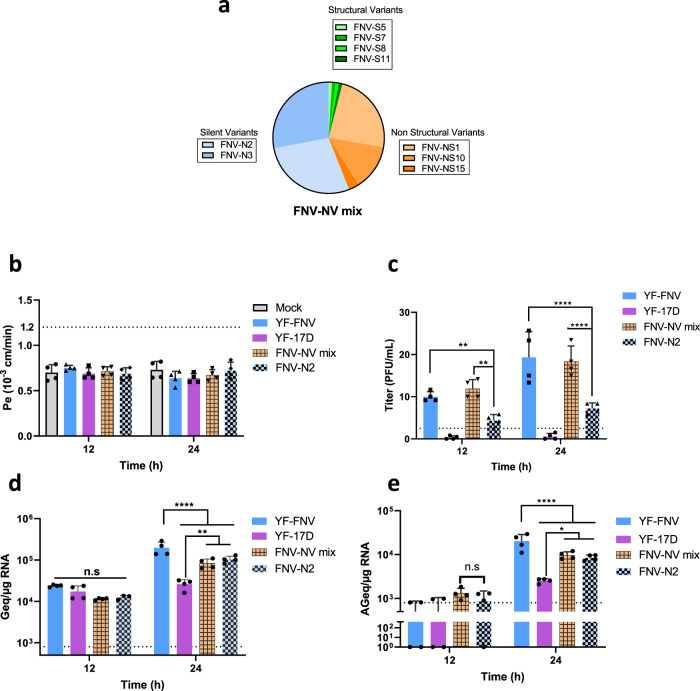
Reconstitution of the genetic diversity of YF-FNV contributes to its neuroinvasiveness. **a** Schematic representation of the composition of the FNV-NV-mix population formed by nine FNV-N structural variants (FNV S5,−7, −8, and −11), non-structural (FNV NS1, −10 and −15), and silent variants (FNV-N2 and −3). **b**–**e** Comparision of neuroinvasiness of FNV-N mix population to those of parental YF-FNV. hCMEC/D3 cells were inoculated with a 3500 PFU of FNV-N mix. **b** Quality control of the endothelial permeability. **c** The amounts of infectious particles released in the lower chamber were assessed at 12 and 24 hpi by viral titration. Titers were expressed as plaque forming unit per milliliter (PFU/mL). **d**, **e** The relative amounts of cell-associated viral genomic RNA (**d**) and antigenomic RNA (**e**) of each virus were determined by RT-qPCR analysis at 12 and 24 hpi. Amounts of viral RNA were expressed as genome equivalents (Geq) or antigenome equivalents (AGeq) per microgram of total cellular RNA. Statistical test: Two-way ANOVA with post hoc Sidak’s test (n.s. not significant; *p < 0.05; ****p < 0.0001). Data in all bar graphs were means ± SD of four independent experiments (*N* = 4) performed in triplicate (*n* = 3). Dashed lines indicate the limit of detection.

We reconstituted a viral stock in which nine YF-FNV variants were mixed (YF-FNV-NV-mix) at the same frequency than in the original stock (Fig. 4a) and compared the neuroinvasiveness of this reconstituted viral population with those of YF-FNV and YF-17D strains used respectively, as positive and negative controls of neuroinvasivness in these experiments and those of FNV-N2 as a poorly diversified variant that exhibits the same consensus sequence as YF-FNV (Fig. 4b, c). Monolayers of hCMEC/D3 cells were inoculated with 3500 PFU with the four viral stocks. Cells and bottom chamber media were collected at 12 and 24 hpi. The integrity of the hCMEC/D3 cell monolayer remained intact over the course of the four infections (Fig. 4b). Similar amounts of infectious particles were recovered from basolateral membranes of cells infected with YF-FNV-NV-mix and YF-FNV, both at 12 and 24 hpi (Fig. 4c). As expected from previous results (Fig. 3c), hCMEC/D3 cells infected with FNV-N2 released significantly less infectious particles than YF-FNV at these two time points (Fig. 4c). RT-qPCR analysis show that the amount of genomic plus- and minus of FNV-N mix RNA detected intracellularly increased significantly from 12 to 24 hpi (Fig. 4d, e), demonstrating that YF-FNV-NV-mix replicates efficiently in hCMEC/D3 cells. YF-FNV-NV-mix, FNV-N and FNV-N2 produced around 10^5^ copies of genomic viral RNA and 10^4^ copies of minus strand RNA per μg of total RNA at 24 hpi, revealing that the three viruses replicated in hCMEC/D3 cells with similar efficiency (Fig. 4d, e). Together, these data strengthen our conclusion that viral genetic diversity contributes to the neuroinvasion of YF-FNV.

## Discussion

By using a mBBB, we showed that YF-FNV uses two means to cross the mBBB: (1) Early times after virus inoculation in the upper compartment (8 and 12 h), virus particles cross the mBBB without disrupting the barrier. This mechanism is independent of replication of the virus, an event which occur between 12 and 24 h (YFV cycle is estimated to be around 18–20 h^39^), (2) in addition, the mBBB passage of YF-FNV is also observed and amplified once the virus starts to replicate in endothelial cells without increasing the mBBB permeability. The BBB passage associated with replication has frequently been observed for neurotropic flaviviruses, including Japanese encephalitis virus (JEV)^40–42^, West Nile virus (WNV)^43^, ZIKV^44–48^, DENV^49^, and Tick-borne encephalitis virus (TBEV)^50^. In contrast, the BBB passage by neurotropic flaviviruses before replication is less firmly documented (WNV^51^; JEV^52^; ZIKV^53^). Transcytosis is a biological process taking place in a lot of different cell types including neurons, intestinal cells, osteoclasts, and endothelial cells. In polarized cells, the unidirectional transcytosis transports macromolecules or structures from apical to basolateral plasma membranes, including viruses^53,54^. During this journey, different biological steps, such as endocytosis, intracellular vesicular trafficking, and exocytosis are involved. Endocytosis can be achieved by adsorptive (charge dependent) or receptor-mediated internalization^55,56^. Three types of endocytic vesicles participate in the transport of macromolecules through endothelial cells: clathrin-coated vesicles, caveolin-coated vesicles, and macropinocytosis vesicles^57,58^. Observations of mouse brain sections infected with JEV by electron microscopy has revealed the presence of viruses in endocytosis and transcytosis vesicles, both in endothelial cells and in pericytes^52^. Infectious particles of ZIKV were detected in the lower compartment of a BBB model in the presence of chloroquine, an inhibitor of viral entry that acts by reducing endosome acidity^59^, suggesting that transcytosis may also be involved in the neuroinvasion of ZIKV^60^.

Our data suggest that YV-FNV may reach the CNS via at least two mechanisms: replication and passage through endothelial cells.

Many RNA virus polymerases lack proofreading and correcting activity, which contributes to a high mutation rate during viral replication^61,62^. The short replication cycle of these viruses rapidly generates a heterogeneous viral population, called viral quasispecies, which contains a multitude of variants that can be associated with different phenotypes^33,34,38,61–63^. Different steps of the viral ecology and natural history, such as cell tropism, the requirement to replicate in multiple hosts, and the crossing of biological barriers, constitute a bottleneck which reduces the diversity of viral populations. Such bottleneck leads to an enrichment of some viral variants that exhibit traits that allow them to adapt to the new environment^64^. During YF-FNV infection, the BBB could represent a bottleneck that only neuroinvasive viral particles are able to overcome. We hypothesized that the attenuation process during which YF-FNV was generated, which consisted in 260 repeated passages on mouse brain^7^, could have selected neuroinvasive/neurotropic and neurovirulent variants, thus explaining the higher levels of YEL-AND for the YF-FNV strain compared to the YF-17D strain. Analysis of the diversity of the YF-FNV population after crossing the endothelial cell monolayer at early time postinoculation (YF-FNVn) showed that the passage of the mBBB in a replication-independent manner led to the modification of the frequency of non-synonymous variants in the YF-FNVn subpopulation. We identified a NS1 variant (G53E), which was both present in the initial population and in the YF-FNVn population with a similar frequency (13.1 and 17.4%, respectively). A mutation at this exact position in DENV NS1 (G53D) evolved in the PDK53 strain, which was derived through 53 passages of a wild-type DENV2 16681 strain in primary dog kidney cell^65^. The G53D mutation impaired the glycosylation of NS1 and led to the induction of an ER stress-mediated antiviral response^65^. The NS1 of DENV and ZIKV protein has also been shown to increase the permeability of cultured polarized human endothelial cells isolated from a variety of organs, as well as triggering tissue-specific vascular endothelial dysfunction in mice^66,67^. By contrast, despite binding to human brain microvascular endothelial cells, YF-17D-NS1 was not internalized by these cells and thus did not increase their permeability^66^. The ability of YF-FNV NS1 to modulate the permeability of endothelial cells was not tested. Furthermore, the ability of DENV-NS1 to disrupt the endothelial layers seems to depend on a N-glycosylation site^68^. Therefore, the potential contribution of YF-FNV NS1 glycosylation on the ability of the virus to cross the BBB deserves further investigation.

One of the variants, FNV-S5, was unable to cross the monolayer of endothelial cells prior to viral replication. This variant possesses one mutation in the M protein at position 14 (T14N) and one in the NS1 protein at position 309 (P309S). The M protein is present on the surface of the particle and arises from the cleavage of the precursor protein prM. The prM form, which serves as a chaperone for the E protein, is composed of a N-terminal globular precursor “pr” region (residues 1–91) and an ectodomain M (residues 92–130) connected to the membrane anchor^69^. Several histidine residues of the E and M proteins, such as His216 and His248 of the E, and His7 and His17 of the M protein, have been implicated in the accessibility of the fusion loop of E protein^70–72^. Histidine at position 6 of the M protein is important for the maturation and infectivity of DENV particles^73^. As FNV-S5, FNV-S11 has a mutation in the ectodomain of the M protein, at position 6 (T6M). These mutations, which are in close proximity to the mentioned histidine residues, may modify the sensitivity of the protein to endosomal pH, thus altering the transcytosis of the viral particles towards the brain compartment. FNV-S7 possesses a mutation in the E envelope protein at position 268 (T286A). This mutation, which is positioned in a histidine-rich structural pocket that binds to the ectodomain of the M protein^70^, renders FNV-N7 poorly replicative in brain endothelial cells. The interface between M and E proteins seems thus to be key for YF neuroinvation.

**Fig. 5:**
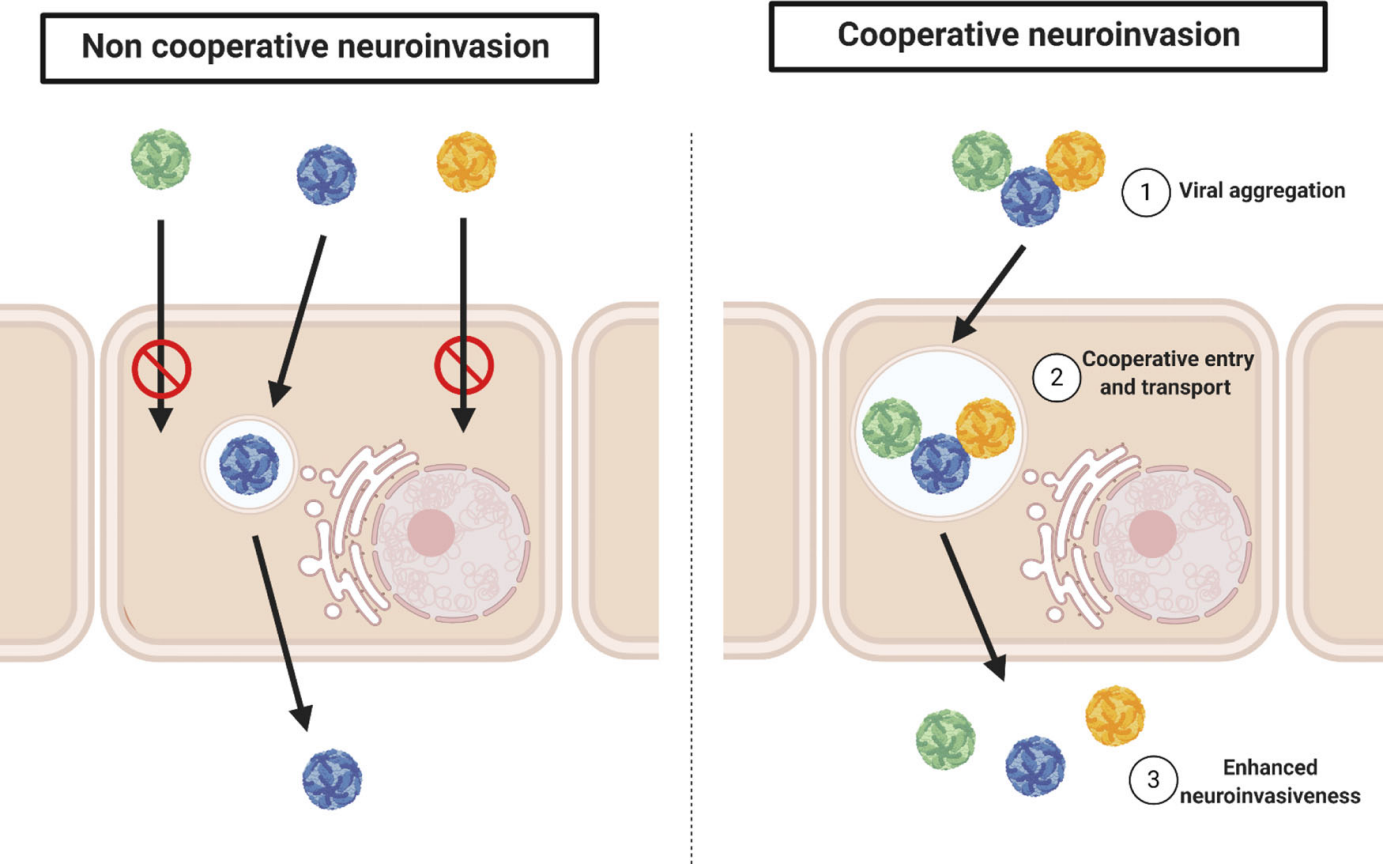
Cooperation between YF-FNVn variants to form collective structure to cross the BBB. **(Left panel**) Non cooperative neuroinvasion: schematic representation showing the passage of a homogeneous population of virions through the BBB. Two non-neuroinvasive viruses (yellow and green) cannot cross the endothelial barrier while the blue neuroinvasive virus crosses. (**Right panel**) Cooperative neuroinvasion: schematic representation showing the viral cooperation mechanism. During viral production, viral aggregates (1) may be formed allowing the concentration of different viruses on the cell surface. The neuroinvasive blue virus will allow non-neuroinvasive viruses (yellow and green) to enter and cross the endothelial cells of the BBB (2) This mechanism would increase the amount of viral particles crossing the BBB (3) The figure was generated using Biorender software.

No significant difference between the parental and the consensus sequence of YF-FNVn was detected, suggesting that the majority of the variants that have crossed the mBBB possess the parental sequence. This implies that the sequence of YF-FNV alone is, in principle, sufficient to cross the mBBB by passage through the endothelial cells. However, our data also showed that the plaque-purified variants exhibited an altered neuroinvasion by both passage through and replication in the endothelial cells, as compared to YF-FNV. Moreover, the FNV-N2 variant, which differs from YF-FNV exclusively by silent mutations, exhibited reduced ability to cross the endothelial cell monolayer, both in a replication-dependent and -independent manner, highlighting the importance of the genomic diversity for YF-FNV neuroinvasion. Reconstitution of the viral diversity by mixing plaque variants emphasizes further the key role of the viral diversity for mBBB crossing. How can we explain that a polyclonal (heterogeneous) YF-FNVn population crosses them BBB more efficiently than a homogenous population? The most likely explanation is that YF-FNV variants cooperate to cross the mBBB. Such an event could result from gathering several variants into a structure allowing a collective entry or passage through the endothelial cells (Fig. 5). Viral aggregates or pools of virions associated or not with cellular debris, or packed in exosomes, could be present in viral stocks. Indeed, viral aggregates have been documented for several viruses such as HTLV-1, HIV-1, Enteroviruses, HAV, Rotaviruses, Noroviruses, Baculoviruses, ZIKV, and VSV^74,75^. Such event can lead to an enhanced infectivity through a mass effect, which can be a major advantage for delivering numerous virions into the CNS avoiding in part the BBB protective effect, and could also be responsible for the sustainability of the genetic diversity of the virus and its short-term viral fitness^76^. In view of this hypothesis, it is interesting to note that deep sequencing analysis proved that FNV exhibits a considerable population diversity which is not shared by other YFV vaccine strains^38^ and which reflect that YF-FNV population can not be stably fixed on the contrary of YF-17D. Nevertheless, these viral aggregates could be delivered by passing through the endothelial cell barrier. Endothelial cells are indeed prone to phagocytosis and they are capable of engulfing large structures such as bacteria, apoptotic cell bodies, myelin debris, and even non biological structures like latex particles^77–80^. Furthermore, pathogens can travel through the endothelial cells by a transendothelial process as it has been shown for bacteria^81,82^ and viruses (Coxsackievirus^83^, Echovirus^84^, Mumps^85^, and LCMV^86^). The reality of these viral aggregates and the transendothelial passage of the mBBB should be further studied.

Furthermore, YEL-AND may also be caused by single NV. Indeed, such variant, which carried two mutations in the E protein, has been isolated from a fatal case of YF-17D-associated human encephalitis^18^. However, it is not excluded that this type of variant reached the CNS by means of viral cooperation. Thus, as a precaution to not select for neuroinvasive single variants, or pool of variants, it is crucial to follow the WHO recommendations and not vaccinate children under 2 years of age with fractionated vaccine doses^87^.

Limited genetic diversity appears to contibute to the safety profile of live-attenuated vaccine^88^. Experimental vaccine candidates have also been obtained by restricting the evolutionary potential of viruses as a strategy to attenuate them^89,90^. Our work suggests that the low diversity of YF-17D reduces cooperation among viral variants, which may make it less prone to cross the BBB and causes YEL-AND than YF-FNV. Assessing viral quasispecies and their evolution in live-attenuated vaccines may guide the development of safer vaccines.

## Methods

### Cells and reagents

The human brain endothelial cell line (hCMEC/D3), obtained from Cedar Lane, Canada, through an MTA agreement, was maintained at 37 °C on rat type I collagen (R&D Systems, #3443-100-01)-coated flasks in EndoGro medium (Merck Millipore, SCME004) supplemented with 5% heat-inactivated fetal bovine serum (FBS) (Gibco, #11573397) and 1% penicillin/streptomycin (P/S) (Thermofisher, #15140). Vero cells (African green monkey kidney epithelial cells) obtained from the American Type Culture Collection (ATCC, #CRL-1586) were cultured at 37 °C and 5% CO2 in Dulbecco’s Modified Eagle Medium (DMEM) containing GlutaMAX (Thermo Fisher Scientific) and supplemented with 5% FBS and 1% P/S. Lycorine, an alkaloid with virucidal effect (BIOMOL Research Laboratories) was dissolved in DMSO and used at a 3 μM concentration. Mock-treated reactions were incubated with 1% DMSO without lycorine.

### Viral strains and UV treatment

The FNV vaccine strain (YF-FNV) is a gift of Dr Amadou Alpha Sall from the virus collection of the Institut Pasteur in Dakar, Senegal (GeneBank MG051218.1). The virus was reconstituted from a vaccine vial (batch 9404, #110494) used for mass vaccination in Africa^9^. The YF-17D vaccine strain (YF-17D 204 [batch year 2001] STAMARIL, Sanofi Pasteur, Lyon; GeneBank MG051217.1) was provided by the Institut Pasteur Medical Center, Paris. Viral stocks were produced by infecting Vero cells grown in EndoGro medium supplemented with 2% FBS and 1% P/S at a multiplicity of infection (MOI) of 0.1. Supernatants were collected 4 days later, centrifuged for 5 min at 1200x*g* to remove cell debris, aliquoted and stored at −80 °C. After four passages, the two viral stocks were analysed by NGS (Supplementary Table 2). Viruses were inactivated by exposure to UV light (4.75 J/cm2) for 30 mins at a distance of 10 cm.

### Preparation of the mBBB, endothelial permeability assay, and quality controls

The preparation of mBBB was undertaken as already described^91^. Briefly, monolayers of hCMEC/D3 were grown on Transwell^®^ inserts (Corning). After 6 or 7 days, the cells reached confluence (around 500,000 cells) and were inoculated with the viral strains. Then, they were transferred into chambers of 24-well cell culture plate containing 1.5 mL of transport medium (HBSS, Thermofisher Scientific, #14025-100) supplemented with 10 mM hepes buffer (Thermofisher Scientific, #15630-080), and 1 mM sodium pyruvate (Thermofisher Scientific, #11360). Quality control of endothelial permeability was performed using the Lucifer yellow (LY) permeability assay. 0.5 mL of medium containing 50 μM of fluorescent LY (Merck, #L0259) was added to the upper compartment. After 10, 25, and 45 min, the insets were transferred to new wells, previously filled with 1.5 mL transport medium. After 45 min, aliquots were taken for each time point, from both lower and upper compartments and the LY concentration was determined using a VarioScan LUX fluorescence spectrophotometer (Thermofischer Scientific, #VL0L0TD0) (excitation: 430 nm, emission: 540 nm). The endothelial permeability coefficient (P_e_) of LY was calculated as described by Siflinger-Birnboim et al.^92^. The permeability of the insert only (PSf) and the permeability of the insert plus endothelial cells (PSt) were taken into consideration using the following equation: 1/PSe = 1/PSt − 1/PSf. The value of the permeability of the endothelial monolayer was then divided by the surface area of the porous membrane of the insert to obtain the P_e_ of the molecule in cm/min^−1^. We considered that an endothelial barrier with a P_e_ greater than 1.2 has a compromised integrity^23^ and therefore was not used for any experiment. The quality of the mBBB was also assessed by transendothelial electrical resistance (TEER) as described previously^93^, as well as by the presence of the tight junctions proteins Zonula Occludens-1 (Z0-1) and Cadherin. Immunostaining was conducted on the hCMEC/D3 coated insert filters, as described previously^27^ using anti-ZO-1 (Thermofisher Scientific, #61-7300) and anti-pan cadherin (Merck, C1821) antibodies. Goat-anti-rabbit-alexa 488 (Thermofisher Scientific, #A11034) and goat-anti-mouse-alexa488 (Thermofisher Scientific,#A-11001) were used as secondary antibodies. The inserts were mounted under coverslip with ProLong™ gold antifade reagent (Thermofischer Scientific, #P36930) and the cells were imaged with a Leica DM 5000B UV microscope equipped with a DC 300FX camera. Alterations of the mBBB was undertaken with hyperosmotic 1 M mannitol treatment (Merck, #M9546) for 1.5 h as described previously^27^. Wounding of the mBBB was conducted by gentle scratching of the endothelial cell monolayer on insert filters with a 10 µl micropipette tip. The presence of the scratch on the cell monolayer and the integrity of the porous cell culture polyester membrane was evaluated by phase contrast microscopy.

### Viral titration by plaque assays

Vero cells seeded in 24-well plates were incubated with 10-fold serial dilutions of viral supernatants in DMEM-Glutamax I supplemented with 2% FBS and 1% P/S for 2 h at 37 °C.Viral inoculum were removed and replaced by 800 μL of a 3:1 mixture of DMEM-Glutamax I medium supplemented with 2% FBS and a 1.6% carboxymethylcellulose (CMC) solution (VWR, low viscosity #276494), containing 4.25 mg/mL NaCl. After incubation for 5 days at 37 °C and 5% CO2, the CMC was removed, and the cells were washed twice with PBS, fixed with a solution containing 4% paraformaldehyde, 5% crystal violet, and 150 mM NaCl for 20 min, then rinsed two times with water. Plaques were counted in each well to determine the viral titer. Viral titer was expressed as the number of plaque forming units (PFU) per milliliter of supernatant (PFU/mL).

### RNA extraction and RT-qPCR analysis

Total RNAs were extracted from cell lysates using the NucleoSpin RNA plus kit (Macherey-Nagel, #740984). Total cDNA synthesis was performed with an equal amount of purified RNA (0.25 μg) using the SuperScript III reverse transcriptase (Invitrogen, #18080044). Random primers (Roche, #11034731001) were used for the detection of positive-sense RNA and an NS5-specific primer (YF-NS5-AntiG 5′-GGCGGTGAGTGAGACGAC-3′) was used for the quantification of the viral negative-strand (or antigenome). Quantitative real-time PCR was performed on a real-time PCR system (QuantStudio 6 Flex, Applied Biosystems) with SYBR Green PCR Master Mix (Thermofisher Scientific, #4367659). Data were analyzed with the ΔΔCT method, with all samples normalized to GAPDH. Primers used in this study were: GAPDH (GGTCGGAGTCAACGGATTTG and ACTCCACGACGTACTCAGCG) and YF-NS5 (ATGGCAGGAGGATTGTGGTG and GTTGTGCGTCCTTGTGGAAC). Viral genomes equivalent (Ge) and viral antigenomes equivalent (AGe) were determined by extrapolation from a standard curve generated from serial dilutions of the plasmid encoding the YF-R.luc2A-RP^94^ and are expressed as Ge/AGe for 1 μg of total RNA (Geq/μg of RNA or AGeq/µg of RNA).

### Plaque purification of clonal NV

The upper compartment of mBBB of hCMEC/D3 grown on Transwell^®^ inserts were inoculated with 3500 PFU of YF-FNV. The medium from the lower compartments corresponding to the YF-FNVn subpopulation of FNV was harvested at 8 and 12 hpi. A fraction of the samples was used to titer the YF-FNVn subpopulation and another fraction (500 µl) was amplified on Vero cells by a 4 day-passage. Vero cells monolayers were infected with serial dilutions of the amplified YF-FNVn stock and incubated in a semi-solid medium with 2% agarose. Plaques were assumed to be clonal and were harvested at 8 and 12 hpi with a pipette tip and amplified on Vero cells cultured in EndoGro medium supplemented with 2% FBS and 1% P/S.

### Deep sequencing of viral stocks and YF-FNVn population

Viral RNAs were extracted using the NucleoSpin RNA plus kit (Macherey-Nagel, #740984), following the manufacturer’s recommendations. The RNAs were eluted in RNAse-free water and treated with DNAse before being stored at −80 °C. cDNA synthesis was performed with the SuperScript^®^ III RT-PCR system kit (Invitrogen, #18080044). Three fragments of the viral genome (a, b, c), ranging in size from 3725 to 3891 bp, were amplified using the Phusion High-Fidelity DNA Polymerase (NEB, #M0530S/L) kit using primers described previously^95^. The PCR products were analyzed on a 1% agarose gel and purified with the QIAquick PCR Purification kit (Qiagen, #28104) resuspended in RNAse-free water and stored at −20 °C. Libraries were prepared after pooling 400 ng of the three overlapping amplicons. The three amplicons were fragmented into 800 bp length products. The Illumina libraries were prepared with the Nextera^®^ XT DNA kit (Illumina, FC-131-1024) using 0.2 ng/μL of the three fragmented amplicons. The Nextera^®^ XT Index Kit (Illumina, #FC-131-2001) oligo multiplexes were used. Qubit BR dsDNA analysis kit (Thermofisher Scientific, #10625443) was used for quantification. Samples from the library diluted to 4 nM and were sequenced on a MiSeq 500 sequencer (Illumina) with the MiSeq Nano V2 (300 cycles) kit (Illumina, #MS-102-2002), to generate paired-end reads of 150 nt. The PhiX control library was used for quality control and calibration in the sequencing cycles (Illumina, #FC-110-3001).

### Sequence analyses and comparison of diversity of viral subpopulations

The analysis was performed using the CLC Genomics Workbench software version 8.5 (CLC bio-Qiagen, #832022). The analytical pipeline consists in quality control of the run, trimming of low-quality reads, reads assembly on the target reference genome of YF-FNV or YFV-17D (respectively, Genbank MG51218.1 and MG051217.1) and low frequency variant calling. All algorithms are proprietary of CLC Genomics Workbench and are described in the user manual (chapters 25 to 29). As expected for short viral genomes, a high sequencing depth was obtained. The parameters used at each step were selected to maximize stringency (Supplementary Note 1). The complete set of NGS data has been filed to the NCBI SRA database under the PRJNA675082 accession number.

### Reconstitution of a viral population with individual FNV clones

Individual viral stocks were made for FNV-S5, FNV-S7, FNV-S8, FNV-S11, FNV-NS1, FNV-NS10, FNV-NS15, FNV-N2, and FNV-N3. All stocks were titrated on Vero cells. The YF-FNV mix was reconstituted by mixing these nine individual variants by respecting the ratio observed in the original stock, as follow: FNV-S5 (x1), FNV-S7 (x1), FNV-S8 (x1), FNV-S11 (x1), FNV-NS1 (x23.8), FNV-NS10 (x13.2), FNV-NS15 (x3), FNV-N2 (x23), and FNV-N3 (x28).

### Statistical analysis

The statistical analyses were performed using GraphPad Prism 8.4.2 software for Windows. Mann–Whitney *U* test was used for the comparison of mean and median between two groups for the SNV of the NGS analysis. For comparison between multiple groups (titration and RT-qPCR analysis), two-way analysis of variance (ANOVA) with post hoc Sidak’s multiple comparison tests were used. *P* values <0.05 were considered significant, (*p < 0.05; **p < 0.01; ***p < 0.001; ****p < 0.0001; ns not significant).

### Reporting Summary

Further information on research design is available in the Nature Research Reporting Summary linked to this article.

## Supplementary information

Supplementary Information

Reporting Summary

## Data Availability

The complete set of NGS data has been uploaded to the NCBI SRA database under the PRJNA675082 accession number. The viral variants generated during this study are available from the corresponding authors upon request and by Material Transfer Agreement.
