# Supplementary material for: Genomic diversity contributes to the neuroinvasiveness of the Yellow fever French neurotropic vaccine
Source: NPJ Vaccines. 2021 Apr 26;6:64. doi: 10.1038/s41541-021-00318-3 (PMC8076279; doi:10.1038/s41541-021-00318-3)
Supplement: Supplementary file 1 — Supplementary Information [file 41541_2021_318_MOESM1_ESM.pdf]

SUPPLEMENTARY FIGURES

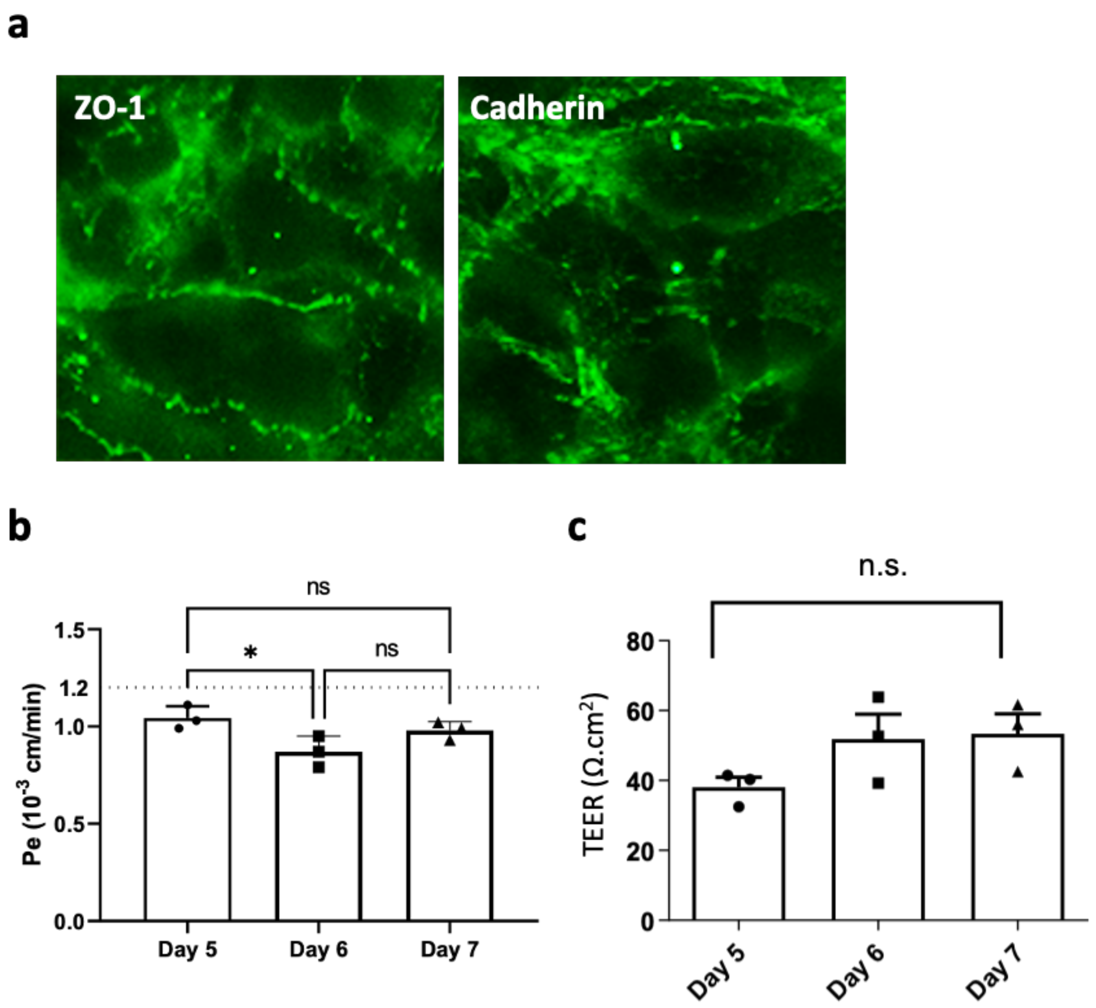

Supplementary Figure 1. Assessment of the mBBB integrity overtime. (**a**) Z0-1 and Cadherin expression on mBBB was examined at day 5 post-seeding by microscopy using anti-Z0-1 and Cadherin antibodies (green). Images are representative of two independent experiments. (**b**) Functional integrity of the mBBB was controlled by quantifying the transport of the small fluorescent molecule Lucifer Yellow (LY) at different days post-seeding. Statistical test: one way ANOVA with Bonferroni post-hoc test, ns=non statistical, *=P 0.0477. (**c**) Trans-endothelial electrical resistance (TEER) values, expressed in Ohms (Ω/cm2), were collected at different days post-seeding. Statistical test: one way ANOVA plus Tukey post-hoc test, ns=non statistical. Results are representative of three independent experiments using duplicate Transwells.

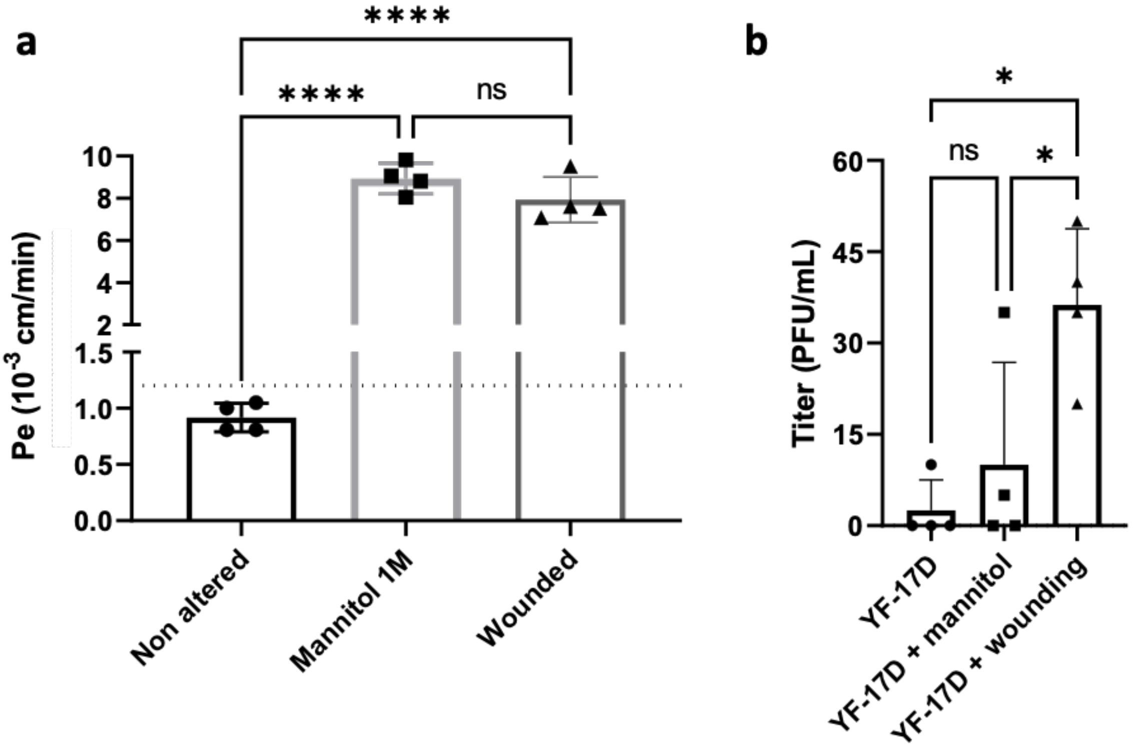

Supplementary Figure 2. A disrupted mBBB allows for an enhanced passage of virions as compared to a non-altered mBBB. The upper compartment was inoculated with 3,500 pfu of YF-17D. (**a**) The integrity of the mBBB was quantified by LY transport post treatment with mannitol (1M for 90 min), wounding or no treatment. The threshold limit for a mature mBBB with a proper physiological status was set at a permeability of $1.2x10^{-3}$ cm/min (dotted line). **(b**) The amounts of infectious particles released in the lower chamber at 16 hpi were quantified by viral titration and expressed as plaque forming unit (PFU/mL). Statistical test: one-way ANOVA plus Tuckey's post-hoc test *<0.04, **** <0.0001

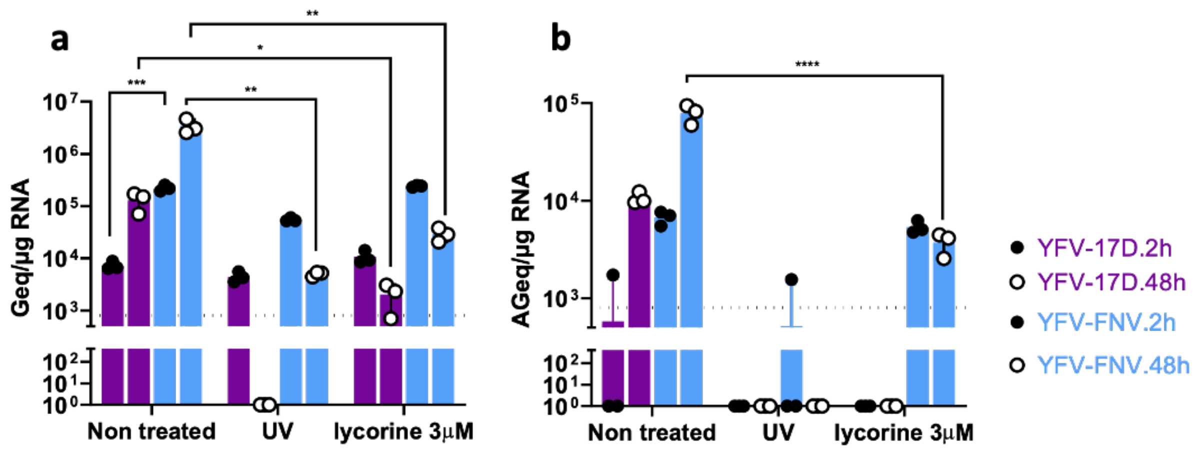

Supplementary Figure 3. Sensitivity of the strand-specific qRT-PCR for detection of negative- and positive-sense RNA transcripts. The relative amounts of genomic (**a**) and antigenomic RNA (**b**) of YF-FNV and YF-17D present in endothelial cells were determined by RT-qPCR analysis at the indicated time. Cells were infected either with replicative viruses or incubated with UV inactivated UV viruses. Cells were either or mock-treated with 1% DMSO or treated with 3 µM of lycorine diluted in DMSO. Amounts of viral RNA were expressed as genome equivalents (Geq) or antigenome equivalents (AGeq) per microgram of total cellular RNA. Statistical test: Two-way ANOVA with post-hoc Sidak's test (n.s: not significant; *p<0.05; ****p<0.0001). The data are representative of three independent experiments performed in triplicate (N=3, n=3).

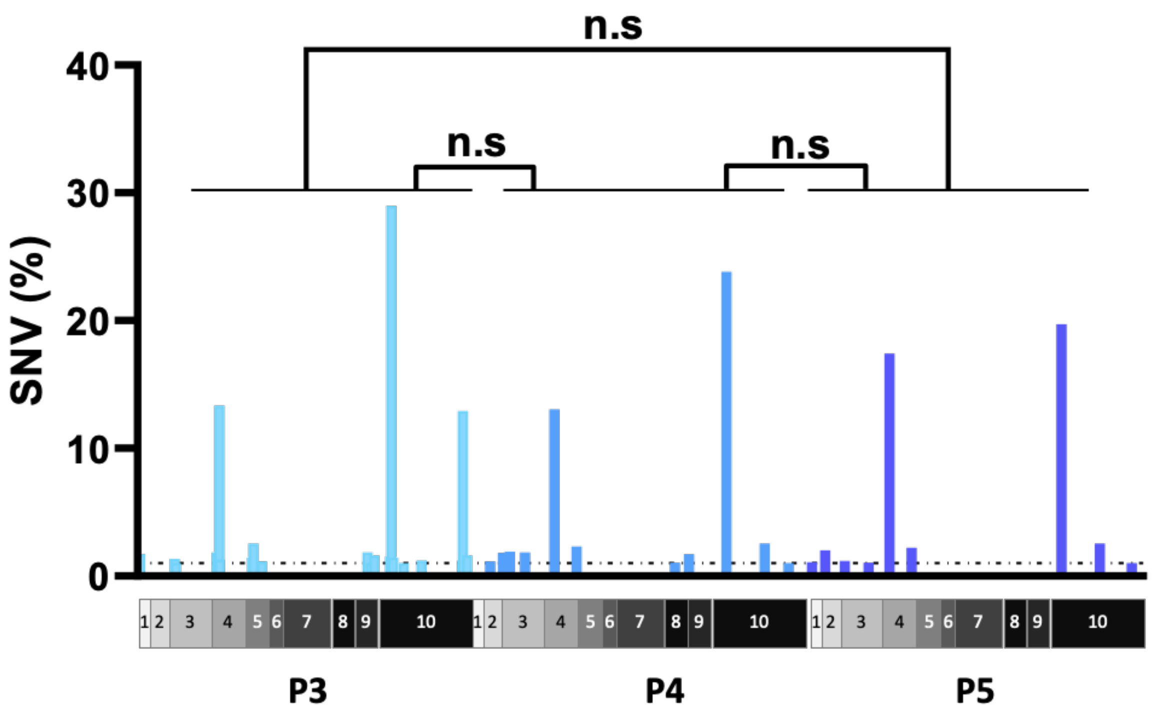

Supplementary Figure 4. YF-FNV genetic diversity is stable in Vero cells. Viral RNAs from YF-FNV stocks corresponding to 4 passages (P2, P3, P4 and P5) in Vero cells were extracted and analyzed by NGS. Coverage depth for these alignments was superior to 500. SNVs and their frequency are represented all along the three consensus sequences. Only the SNVs representing a minimum of 1% of all observations were considered. The analysis revealed that 12 mutations with a frequency> 1% were found in these three passages of YF-FNV, as compared to the reference sequence YF-FNV-P2. When statistically analyzed with the Mann-Whitney U test, no difference in the diversity of the viral populations was observed between the different passages (**$p<0.01$). (n.s: not significant).

SUPPLEMENTARY TABLES

Sequence	nt Position	AA Position	AA reference	AA mutation	FNV P3 variant frequency	FNV P4 variant frequency	FNV P5 variant frequency
5'UTR	87		C	T	1.68	1.95	2.73
prM	128	4	R	S	1.69		1.08
C	554	25	M	V	2.12	1.16	2.0
	968	163	Y	N		1.83	
E	1184	71	N	D	1.35	1.93	1.17
	1676	235	E	*		1.85	
	1947	325	P	L			1.05
NS1	2610	53	G	E	13.34	13.06	17.43
	3318	289	G	D	1.45	2.31	2.21
NS4a	6462	8	V	E		1.04	
NS4b	6911	9	T	S		1.72	
	6912	9	T	N		1.64	
NS5	8100	155	V	A	28.98	23.83	19.73
	9324	563	K	R	1.75	2.54	2.55
	10082	816	V	L		1.02	
3'UTR	10367		T	C	1.18		
	10382		T	C			1.06
	10387		A	G	12.86	15.37	11.57
	10523		A	G	1.55	4.43	1.16

Supplementary Table 1. Three passages on Vero cells had no or very little impact on the diversity of the FNV population. NGS analysis were used to determine the consensus sequence of the YF-FNV population after three passages in Vero cells.

Protein	nt Position	AA Position	AA FNV-P4	AA 17D-P4	% Identity (per protein)
C	357	80	A	V	98.3
	387	90	G	S	
prM	555	25	M	V	99.4
E	993	7	A	T	96.8
	1128	52	G	R	
	1134	54	V	A	
	1140	56	A	V	
	1431	153	K	N	
	1482	170	A	V	
	1491	173	T	I	
	1572	200	K	T	
	1719	249	D	N	
	1869	299	M	I	
	1887	305	S	F	
	1947	325	P	S	
	2112	380	T	R	
	2193	407	A	V	
	2220	416	A	T	
	2343	457	I	M	
NS1	2688	79	L	F	99.4
	3372	307	I	V	
NS2a	3558	17	L	M	97.4
	3753	82	V	I	
	3822	105	A	T	
	3861	118	M	V	
	4008	167	T	A	
	4014	169	L	F	
	4023	172	T	A	
	4056	183	S	F	
	3990	161	I	L	
NS2b	4359	60	A	S	98.5
	4506	109	I	L	
NS3	4608	13	V	I	99.2
	5145	192	K	M	
	5154	195	I	V	
	5409	280	I	V	
	6024	485	D	N	
NS4a	6528	30	L	F	99.2
2K	6843	9	F	Y	91.3
	6876	20	V	A	
NS4b	6942	19	S	L	98.4
	7179	98	I	V	
	7380	165	V	A	
	7581	232	Y	H	
NS5	7641	2	T	S	99.0
	7701	22	Q	R	
	8409	258	T	I	
	8640	335	K	R	
	9228	531	L	F	
	9615	660	R	K	
	10143	836	E	K	
	10269	878	V	I	
	10338	901	P	L	

Supplementary Table 2. Characterization and comparison of the two viral strains used in the study. YF-17D and YF-FNV stocks were produced by infecting Vero cells at a MOI of 0.1. After 4 passages, the 2 viral stocks were analyzed by NGS.

SUPPLEMENTARY NOTE 1

NGS analysis pipeline in CLC Genomics Workbench v8.5

A high stringency was applied to all parameters as the target sequence is short for an NGS sequencing approach, leading to an extremely high coverage of the target genomes. Applying stringent parameters still lead to a quite high genome coverage and sequencing depth.
No data subsampling was done.

Data import
Raw data import from Illumina using Illumina paired imported in FASTQ format
<u>Parameters</u>
Discard read names = No
Discard quality scores = No
Paired orientation = Paired reads (forward-reverse)
Quality score = NCBI/Sanger or Illumina Pipeline 1.8 and later
Remove failed reads = Yes
MiSeq de-multiplexing = No

Run Quality Control:
Includes the following elements:
Total sequences number in data set
Total number of nucleotides in data set
Numbers of paired sequences

Reads trimming
<u>Parameters</u>
Ambiguous trim = Yes
Ambiguous limit = 0
Quality trim = Yes
Quality limit = 0.01
Create report = Yes
Save discarded sequences = No
Remove 5' terminal nucleotides = No
Maximum number of nucleotides in reads = 1,000
Minimum number of nucleotides in reads = 50
Discard short reads = Yes
Remove 3' terminal nucleotides = No
Discard long reads = Yes
Save broken pairs = No

Map reads to reference
<u>Reference sequence</u> :
Yellow fever virus strain French Neurotropic Virus (FNV) Genbank ID : MG051218.1
<u>Mapping parameters</u>
References = YFV_FNV
Masking mode = No masking
Mismatch cost = 2
Cost of insertions and deletions = Linear gap cost

Insertion cost = 3
Deletion cost = 3
Insertion open cost = 6
Insertion extend cost = 1
Deletion open cost = 6
Deletion extend cost = 1
Length fraction = 0.98
Similarity fraction = 0.98
Global alignment = No
Auto-detect paired distances = Yes
Non-specific match handling = Ignore
Output mode = Create stand-alone read mappings
Create report = Yes
Collect un-mapped reads = Yes

Variant calling
The variant detection algorithm is based on LoFreq[1], called Low Frequency Variant Detection in CLC Genomics Workbench

Parameters

Neighborhood radius = 5
Maximum gap and mismatch count = 2
Minimum neighborhood quality = 15
Minimum central quality = 20
Ignore non-specific matches = Yes
Ignore broken pairs = Yes
Minimum coverage = 500
Minimum variant frequency (%) = 0.1
Maximum expected alleles = 2
Advanced = No
Require presence in both forward and reverse reads = Yes
Ignore variants in non-specific regions = No
Filter 454/Ion homopolymer indels = No
Create track = Yes
Create annotated table = Yes
Genetic code = 1 Standard

The table is exported into Microsoft excel and additional filters are applied to validate variant calls:

Variant validation parameters

Minimum coverage: 500
Minimum number of variant counts: 5
Frequency threshold: 1%
Forward/reverse ratio: minimum 20%
Average quality: minimum 35 (Phred/Phrap score)

Primers used for YF genome amplification prior to sequencing library preparation:

PCR#	SEQUENCE	L	YF genome primer position start	YF genome primer position end
1	AGTAAATCCTGTGTGCTAATTGAGGTG	27	53	80
	TTGAAAAGGCAGCAATCAACGC	22	3703	3725
2	GGGTTACAGCTGGAGAAATACATGC	25	3498	3520
	TGCTGCGCTTTCATTCCAGGTA	22	7256	7278
3	TGCTGGAGAAAACCAAAGAGGA	22	6900	6918
	GGTCTTTCCCTGGCGTCAATA	21	10750	10768

Primer sequences were not excluded from genomes assemblies but it was checked that none of the variants called were located within any primer sequence.

1. Wilm, A. *et al.* LoFreq: A sequence-quality aware, ultra-sensitive variant caller for uncovering cell-population heterogeneity from high-throughput sequencing datasets. *Nucleic Acids Res.* **40**, 11189–11201 (2012).
