# Supplementary material for: Genomic diversity contributes to the neuroinvasiveness of the Yellow fever French neurotropic vaccine
Source: NPJ Vaccines. 2021 Apr 26;6:64. doi: 10.1038/s41541-021-00318-3 (PMC8076279; doi:10.1038/s41541-021-00318-3)
Supplement: Supplementary file 2 — Reporting Summary [file 41541_2021_318_MOESM2_ESM.pdf]

nature research

Corresponding author(s): Nolwenn Jouvenet

Last updated by author(s): 01-12-2020

Reporting Summary

Nature Research wishes to improve the reproducibility of the work that we publish. This form provides structure for consistency and transparency in reporting. For further information on Nature Research policies, see our Editorial Policies and the Editorial Policy Checklist.

Statistics

For all statistical analyses, confirm that the following items are present in the figure legend, table legend, main text, or Methods section.

n/a	Confirmed	
☐	☒	The exact sample size (*n*) for each experimental group/condition, given as a discrete number and unit of measurement
☐	☒	A statement on whether measurements were taken from distinct samples or whether the same sample was measured repeatedly
☐	☒	The statistical test(s) used AND whether they are one- or two-sided<br>*Only common tests should be described solely by name; describe more complex techniques in the Methods section.*
☒	☐	A description of all covariates tested
☐	☒	A description of any assumptions or corrections, such as tests of normality and adjustment for multiple comparisons
☐	☒	A full description of the statistical parameters including central tendency (e.g. means) or other basic estimates (e.g. regression coefficient) AND variation (e.g. standard deviation) or associated estimates of uncertainty (e.g. confidence intervals)
☒	☐	For null hypothesis testing, the test statistic (e.g. *F*, *t*, *r*) with confidence intervals, effect sizes, degrees of freedom and *P* value noted<br>*Give P values as exact values whenever suitable.*
☒	☐	For Bayesian analysis, information on the choice of priors and Markov chain Monte Carlo settings
☒	☐	For hierarchical and complex designs, identification of the appropriate level for tests and full reporting of outcomes
☒	☐	Estimates of effect sizes (e.g. Cohen's *d*, Pearson's *r*), indicating how they were calculated

Our web collection on statistics for biologists contains articles on many of the points above.

Software and code

Policy information about availability of computer code

Data collection	n/a
Data analysis	Prism -GraphPad Software Inc. version 8.4.2<br>CLC Genomics Workbench software version 8.5

For manuscripts utilizing custom algorithms or software that are central to the research but not yet described in published literature, software must be made available to editors and reviewers. We strongly encourage code deposition in a community repository (e.g. GitHub). See the Nature Research guidelines for submitting code & software for further information.

Data

Policy information about availability of data

All manuscripts must include a data availability statement. This statement should provide the following information, where applicable:

- Accession codes, unique identifiers, or web links for publicly available datasets
- A list of figures that have associated raw data
- A description of any restrictions on data availability

The data that support the findings of this study are fully available from the corresponding author upon request.

Field-specific reporting

Please select the one below that is the best fit for your research. If you are not sure, read the appropriate sections before making your selection.

☒ Life sciences ☐ Behavioural & social sciences ☐ Ecological, evolutionary & environmental sciences

For a reference copy of the document with all sections, see nature.com/documents/nr-reporting-summary-flat.pdf

Life sciences study design

All studies must disclose on these points even when the disclosure is negative.

Sample size	Sample size of each experiment was determined by the practical limitations of the protocol which was used. No statistical estimation of sample size was performed.<br>Sequencing experiments were generally performed in biological simplicat or triplicate. The very high correlation between these replicates suggested that this was sufficient.
Data exclusions	For all experiement with our mBBB model, any barrier with a permeability >1.2.10-3 cm/min was excluded.
Replication	Each experiment present in the paper was repeated (between 3 and 4 time per experiment). The next generation sequencing was done once.
Randomization	Samples were not randomized across experiments.
Blinding	Blinding during sample collection and library preparation is not necessary as the output from the sequencing runs is analyzed with an automated pipeline.<br>Additional analyses were performed in an unbiased manner, with pre-established criteria, and by different persons than those who collected and prepared the samples.

Reporting for specific materials, systems and methods

We require information from authors about some types of materials, experimental systems and methods used in many studies. Here, indicate whether each material, system or method listed is relevant to your study. If you are not sure if a list item applies to your research, read the appropriate section before selecting a response.

Materials & experimental systems

n/a	Involved in the study	
☒	☐	Antibodies
☐	☒	Eukaryotic cell lines
☒	☐	Palaeontology and archaeology
☒	☐	Animals and other organisms
☒	☐	Human research participants
☒	☐	Clinical data
☒	☐	Dual use research of concern

Methods

n/a	Involved in the study	
☒	☐	ChIP-seq
☒	☐	Flow cytometry
☒	☐	MRI-based neuroimaging

Eukaryotic cell lines

Policy information about cell lines

Cell line source(s)	Vero cells have been obtained from the American Type Culture Collection ;<br>Human brain endothelial cells (hCMEC/D3) have been obtained from Cedar Lane.
Authentication	The cell lines used were authenticated by the providers
Mycoplasma contamination	All cell lines have been tested negative for mycoplasma contamination
Commonly misidentified lines<br>(See ICLAC register)	No crosscontaminations were reported according to ICLAC for the cell lines used in this study
